# Glandular Segmentation of Prostate Cancer: An Illustration of How the Choice of Histopathological Stain Is One Key to Success for Computational Pathology

**DOI:** 10.3389/fbioe.2019.00125

**Published:** 2019-07-05

**Authors:** Christophe Avenel, Anna Tolf, Anca Dragomir, Ingrid B. Carlbom

**Affiliations:** ^1^CADESS Medical AB, Uppsala, Sweden; ^2^Department of Pathology, Uppsala University Hospital, Uppsala, Sweden; ^3^Department of Immunology, Genetics and Pathology, Uppsala University, Uppsala, Sweden; ^4^Department for Information Technology, Uppsala University, Uppsala, Sweden

**Keywords:** digital pathology, computational pathology, prostate cancer, prostate gland segmentation, histopathological stain, Picrosirius red, hematoxylin

## Abstract

Digital pathology offers the potential for computer-aided diagnosis, significantly reducing the pathologists' workload and paving the way for accurate prognostication with reduced inter-and intra-observer variations. But successful computer-based analysis requires careful tissue preparation and image acquisition to keep color and intensity variations to a minimum. While the human eye may recognize prostate glands with significant color and intensity variations, a computer algorithm may fail under such conditions. Since malignancy grading of prostate tissue according to Gleason or to the International Society of Urological Pathology (ISUP) grading system is based on architectural growth patterns of prostatic carcinoma, automatic methods must rely on accurate identification of the prostate glands. But due to poor color differentiation between stroma and epithelium from the common stain hematoxylin-eosin, no method is yet able to segment all types of glands, making automatic prognostication hard to attain. We address the effect of tissue preparation on glandular segmentation with an alternative stain, Picrosirius red-hematoxylin, which clearly delineates the stromal boundaries, and couple this stain with a color decomposition that removes intensity variation. In this paper we propose a segmentation algorithm that uses image analysis techniques based on mathematical morphology and that can successfully determine the glandular boundaries. Accurate determination of the stromal and glandular morphology enables the identification of the architectural pattern that determine the malignancy grade and classify each gland into its appropriate Gleason grade or ISUP Grade Group. Segmentation of prostate tissue with the new stain and decomposition method has been successfully tested on more than 11000 objects including well-formed glands (Gleason grade 3), cribriform and fine caliber glands (grade 4), and single cells (grade 5) glands.

## Introduction

Digital pathology is an emerging field, where glass tissue slides are scanned and stored as digital images for improved workflow, computer-aided analysis, and storage and management of the data. Digital pathology facilitates remote consultation with experts across the world and may alleviate some of the pathologist deficit that is anticipated in most countries from population growth and increases in disease incidence rates (Weir et al., 2015; Fitzmaurice et al., 2017). Once tissue slides are digitized, computer-aided image analysis makes it possible to enhance the resulting images digitally and also to extract quantitative information to support the pathologist's decision process. Computer-aided analysis has the potential to reduce the intra-and inter-observer diagnostic variation (Allsbrook et al., 2001; Burchardt et al., 2008; Abdollahi et al., 2012) and improve the prognostication, thereby improving the patient's life and reducing the healthcare burden from needless treatment. But computer-aided analysis of tissue data requires high-quality image data, where the tissue components are clearly delineated and where the stain variations and noise are kept to a minimum.

Pathologists rely on multiple, contrasting stains to analyze tissue samples, but histological stains are developed for analysis with a microscope and not for computational pathology applications. In Azar et al. (2013), several different histological stains were evaluated for automatic classification of components in prostate tissue. The stains were tested with both supervised and unsupervised classification methods which showed that some stains consistently outperform others according to objective error criteria.

After selecting one of these stains, we removed one major source of color variations by replacing the tap water, commonly used in histopathological staining protocols, with a bluing agent. Next, a staining protocol that is optimal with regard to staining time and dilution of the stain, counter stain, and bluing agent, was developed to produce a distinct color separation between the two stains. The optimal staining time and dilution was the result of an experimental process that optimized quantitative measures of the distances and the compactness of the color clusters in the Maxwellian color space (Maxwell, 1860; Judd, 1935). The visual results were also deemed by two independent uropathologists as appropriate for diagnosis of digitized images. This methodology yields an optimal stain for separation of stroma and epithelium. However, the segmentation algorithm described below would also work with other stains provided that these stains give a good separation between stroma and epithelium.

In Gavrilovic et al. (2013), the authors developed an automatic method for highly accurate blind color decomposition of histological images into density maps, one for each stained tissue type. The method decouples intensity from color information and bases the decomposition only on the tissue absorption characteristics of each stain. The method also models biochemical noise, as well as noise from the CCD (charge-coupled device) array in the microscope.

Prostate gland segmentation is a key component in automatic malignancy grading and prognostication. From the segmented glands, it is possible to extract features of the architectural pattern that are linked to malignancy and are used by pathologists in routine practice. These features include size and shape of the glands and the luminae, nuclear crowding, and the color of the epithelium. The gland segmentation facilitates ground truth labeling of tissue for both machine and deep learning classification.

In this paper, we illustrate the importance of stain selection, the importance of the development of a staining protocol that creates an optimal color separation between the stain and counterstain, and the importance of the use of a decomposition algorithm that removes intensity variation and acquisition noise for an accurate and robust gland segmentation of prostate tissue.

## Related Work

**Figure 1:**
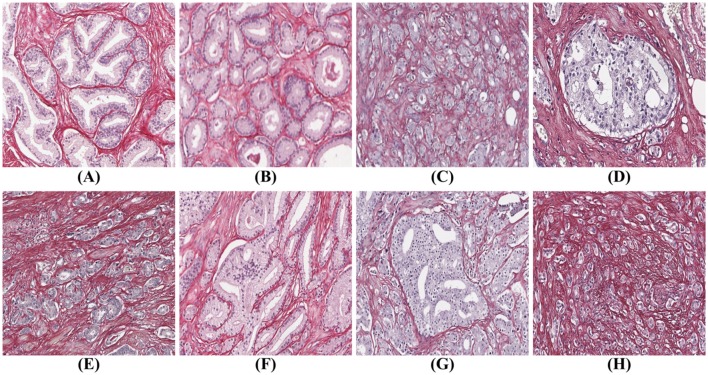
Gleason grades: **(A)** benign; **(B)** well-formed glands (Gleason grade 3); **(C)** poorly formed glands (Gleason grade 4); **(D)** cribriform (Gleason grade 4); **(E)** small fused glands (Gleason grade 4); **(F)** large fused glands (Gleason grade 4); **(G)** intraductal carcinoma (Gleason grade 4); **(H)** poorly formed glands and single cells (Gleason grades 4 and 5).

There are many examples in the literature of prostate gland segmentation as part of automatic malignancy grading systems. Naik et al. (2007) find the lumen using color information and use the lumen boundary to initialize level set curves which evolve until they reach the epithelial nuclei. The final glandular structure only includes the lumen and the epithelium without the nuclei. Nguyen et al. (2012) also start with the lumen and grow that structure to include the epithelial nuclei. Singh et al. (2017) manually annotate gland, lumen, periacinar refraction, and stroma in H&E-stained tissue images, and train a segmentation algorithm on these manual annotations using standard machine learning techniques. The segmentation process continues by region-growing from a seed inside the glands toward the epithelial nuclei. By the authors own admission, the algorithm fails for cribriform glands, since these glands are not lined with epithelial nuclei. Paul and Mukherjee (2016) propose an automatic prostate gland segmentation of H&E-stained tissue images using morphological scale space. The authors assume that glands are surrounded by an epithelial layer where the nuclei appear dark and can be used to delineate the glands. The methods above work on the assumption that a gland is surrounded by a layer of epithelial nuclei, and can thus successfully find only benign glands, glands of Gleason grade (GG) three, and some of poorly formed grade 4, but cannot identify other types, such as cribriform structures and grade 5. Tabesh et al. (2007) use a different approach identifying small objects in the prostate tissue with similar characteristics which are used directly for classification of cancerous and non-cancerous tissue, without identification of the underlying glandular structure. But without the glandular structures it is impossible to identify all the Gleason grades shown in Figure 1.

To automatically identify all glandular patterns illustrated in Figure 1, an algorithm must work from the stromal border and in, not from the center of the gland out. However, traditionally prostatic tissue is stained with hematoxylin and eosin (H&E), which gives poor differentiation between epithelium and stroma, as both stain in shades of red/pink by eosin. A different stain that gives good contrast between glandular epithelium and stroma is required for accurate prostate gland segmentation that works for all types of prostate glands.

While the methods above rely on classical machine learning and image analysis, deep learning has recently generated a great deal of interest for the problem of segmentation and classification of prostate tissue. The first such publication (Litjens et al., 2016), applies convolutional networks (CNN) to prostate tissue analysis. The authors manually delineate cancer regions from H&E-stained prostate tissue and then train a network on patches extracted from these regions. A cancer likelihood map from the CNN shows good agreement with the manual identified cancer regions. The authors also demonstrate that potentially it is possible to automatically exclude a significant portion of benign tissue from the diagnostic process.

In Ing et al. (2018), the authors combine segmentation and classification of glandular regions. They annotate regions in H&E-stained tissue images as stroma, benign glands, Gleason grade 3 and Gleason grade 4&5, and train several public networks with these annotations. The results are compared with manually annotated regions and show a good accuracy for benign tissue, and for high-grade and low-grade cancer. Gummeson et al. (2017) describe the classification of prostate tissue into classes benign, and Gleason grades 3, 4, 5 with a proprietary network. The training dataset is created by cropping tissue images so that each training image contains only one grade. The authors report a high classification accuracy, but the small training dataset may not cover all types of glands.

Jiménez del Toro et al. (2017) describe a completely automatic segmentation and classification method for H&E-stained tissue images. The ground truth is extracted from pathologist's reports in the original diagnoses. The authors propose a method to remove the areas that are not of interest, that is areas in the tissue with few epithelial nuclei, and train public networks on patches in the remaining tissue. The method performs well in separating low grade cancer (Gleason scores 6-7) from high grade cancer (Gleason scores 8-10).

In summary, while deep learning shows great promise for the segmentation and classification of prostate glands, none of the approaches above can segment or classify all types of malignant glands into appropriate categories.

## Materials and Methods

### Prostate Tissue

The prostate tissue used in this study was extracted from whole mount sections from 36 prostatectomies performed at the Uppsala University Hospital, Uppsala, Sweden. The paraffin blocks were cut using a microtome (Microm HM430 microtome from Thermo Scientific, Gerhard Menzel GmbH, Saarbruckener Str 248, D-38116 Braunschweig, Germany), to produce 4 μm thick sections. Each whole mount section was stained with Picrosirius red-hematoxylin (PSR-Htx) (Histolab products AB, Södra Långebergsgatan 36, SE421 32 Västra Frölunda, Sweden), and scanned at 20x with an Aperio AT2 whole slide scanner (Leica Biosystems) and a NanoZoomer S60 Digital slide scanner (Hamamatsu). We tested our segmentation method on 486 images with a resolution of 1500 × 1000 pixels, extracted from the digitized images of whole mounts.

### Malignancy Grading

Prostate tissue is graded according to Gleason (Gleason, 1992; Amin et al., 2003), on a scale from 1 to 5, although in recent years pathologists use only grades 3–5. In the Gleason system, pathologists report the Gleason Score, which in prostatectomy sections is the sum of the grade of the dominant pattern and the grade of the next-most common one, e.g., 3+4 or 4+3, and in biopsies it is the sum of the dominant pattern and the most aggressive pattern, e.g., 3+5.

The International Society of Urological Pathology (ISUP) has proposed an alternative system based on five Grade Groups. The ISUP Grade Groups are based on the proportion of discrete well-formed glands (Gleason pattern 3), cribriform/poorly-formed/fused glands (Gleason pattern 4) and sheets/cords/single cells/solid nests/necrosis (Gleason pattern 5) in the tissue (Epstein et al., 2015).

Prostate cancer malignancy grading relies heavily on the identification of the prostate glands. A benign prostate comprises branched ducts and glands, covered with two types of cells, i.e., acinar and basal cells (Figure 1A). Malignant tumors of low grade are forming glands of regular size with a central lumen, containing only one type of epithelial cells (acinar) with the nuclei located basally (Figure 1B). These individual, discrete well-formed glands are grade 3 on the Gleason scale. When cancer progresses in degree of malignancy, the glands loose uniformity in size and shape and the inter-glandular distance becomes more variable. These glands are referred to as poorly-formed or fine caliber Gleason grade 4 (Figure 1C). Other types of grade 4 glands form cribriform structures with multiple luminae (Figure 1D), glomeruloid structures or fuse into irregular structures (Figures 1E,F). Intra-ductal carcinoma (Figure 1G), which also can form cribriform structures, is currently not graded, but the consensus is to report it and indicate its invariable association with aggressive prostate cancer. Finally, Gleason grade 5 is defined as sheets, chords, files or individual tumor cells (Figure 1H), as well as comedonecrosis in any glands (well-formed or cribriform). In summary all glands have an epithelium with at least one epithelial nucleus, surrounded by stroma.

### Blind Color Decomposition

In Gavrilovic et al. (2013) the authors describe a blind color decomposition method (BCD), that separates the tissue into density maps, each corresponding to one stain/tissue type. The BCD method removes intensity variations present in the samples due to tissue preparation factors, including stain concentration, staining duration, tissue thickness, and fixation, allowing the decomposition to be based only on tissue absorption characteristics. The method transforms the RGB tissue image data into a linear model using the Beer-Lambert law, and then maps the resulting color data to the Beer-Lambert chromaticity triangle (the dual of the Maxwellian chromaticity triangle) (Maxwell, 1860; Judd, 1935), where the distance between two points corresponds to the chromaticity difference between the corresponding colors. Then expectation maximization fits Gaussian distributions to the color data in the Maxwellian plane, from which the reference colors are estimated, which in turn determine the mixing matrices. The relative densities are found using linear decomposition. Finally, noise modeling increases the accuracy of the decomposition further. The BCD method outperforms other color decomposition methods both qualitatively and quantitatively for several types of tissue as demonstrated using ground truth provided by an expert pathologist.

### Picrosirius Red-Hematoxylin

The traditional prostate tissue stain, H&E, which dates from 1896 (Mayer, 1896; Lillie, 1965), does not allow automatic identification of the prostate glandular structure which is key in computer-aided malignancy grading. In Azar et al. (2013), the authors demonstrate that H&E is not ideal for machine learning applications, but that other stains, such as PSR-Htx (Puchtler et al., 1973; Junqueira et al., 1979), perform better, chiefly because the stain of the stroma is distinct from the stain of the epithelium. PSR stains the connective tissue surrounding the glands red, allowing precise identification of the glandular borders, which is required for gland segmentation. Both H&E and PSR-Htx use hematoxylin to stain the nuclear texture.

In the work described herein, we selected PSR-Htx based on the stain comparisons made in Azar et al. (2013). In this study, 13 consecutive tissue sections from radical prostatectomies were stained, each with one of 13 stains, and evaluated for both supervised and unsupervised classification of prostate tissue components. The stains were ranked for supervised classification based on the error rate of non-linear support vector machines and ranked for unsupervised classification by assessing the clustering results of a Gaussian mixture model based on expectation-maximization. PSR-Htx was not the highest-ranking stain, but was selected since it was one of the best performing stains that also gave reproducible results and that did not increase costs over H&E. In Carlbom et al. (2014), the authors show that PSR-Htx is superior to H&E for blind color decomposition into density maps that correspond to the stroma and the epithelium.

**Figure 2:**
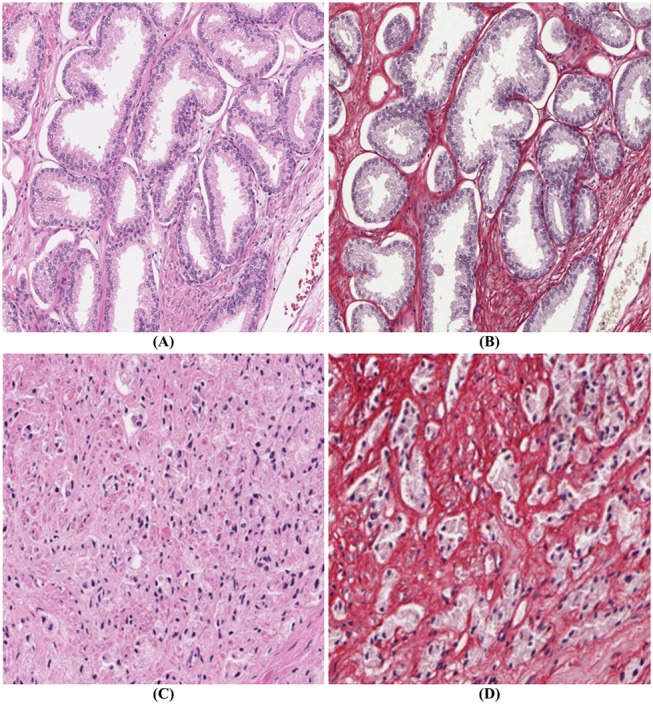
Comparison of PSR-Htx and H&E: **(A)** H&E tissue and **(B)** PSR-Htx tissue, on two consecutive slices of low Gleason grade (3+3); **(C)** H&E tissue and **(D)** PSR-Htx tissue, on two consecutive slices of higher Gleason grade (3+4 focal 5).

PSR-Htx is not only superior to H&E for machine learning applications but in some cases also for visual inspection. This is illustrated in Figures 2A,B, but even more dramatically in Figures 2C,D, that show two consecutive sections, one stained with H&E (C), the other with PSR-Htx (D). In the H&E-stained tissue it is hard to discern any glandular structure which is clearly present in the PSR-Htx-stained tissue. The H&E-stained tissue appears to be of a higher malignancy grade than the tissue stained with PSR-Htx, because no glandular structure appears to be present, while in the PSR-stained tissue, some glandular structures are discernable.

### Development of an Optimal Staining Protocol

The first goal is to determine the optimal Picrosirius red-hematoxylin stain/counter-stain combination, in particular which type of hematoxylin is best suited for color decomposition and in what order PSR and Htx should be applied. We chose to use Mayer's hematoxylin by a regressive method (National Society for Histotechnology, 1973).

The second goal in developing a staining protocol is to reduce the inter- and intra-lab color variations that is noticeable in most publications with H&E stain and that could potentially also be a problem with this new stain. The tap water that is often used to rinse the tissue sample both before and after the hematoxylin staining acts as a bluing agent, changing the reddish purple of the hematoxylin to a blue color. It also decolorizes the tissue if the Picrosirius red is used in a regressive mode. But the quality of the tap water is highly variable, and often tap water leads to inadequate bluing, leaving the tissue with an overall pink color. We replace all tap water rinses with a reliable bluing agent.

**Figure 3:**
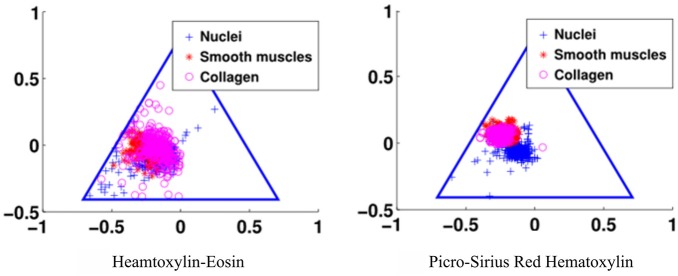
Illustration of the color separation for hematoxylin-eosin, and Picrosirius red-hematoxylin in the Maxwellian triangle.

The third goal for an optimal stain protocol is to choose the staining protocol parameters so that the color absorption in the tissue enables an optimal decomposition of the tissue image into two density maps, one for the epithelium and the other for the stroma. We transform the RGB tissue image data into a linear model using the Beer-Lambert law, and then map the resulting color data to the Beer-Lambert chromaticity triangle (the dual of the Maxwellian chromaticity triangle) (Maxwell, 1860; Judd, 1935). The chromaticity triangle allows us to describe a perfect stain for segmentation: its stain clusters should be compact, separate, and all tissue types should absorb enough stain so that the stain cluster may be detected. In Figure 3, it is clear that the standard stain for prostate cancer, H&E, gives a poor separation of the nuclei from the stroma (smooth muscle and collagen), but the separation for PSR-Htx is very clear.

The staining parameters, staining duration and concentration, are chosen to give optimal separability of the stain clusters in the Beer-Lambert chromaticity triangle and by the amount of color in each cluster. More specifically, the separability is measured quantitatively by the Mahalanobis cluster distance (McLachlan, 1999), and by the Calinski-Harabasz index (Calinski and Harabasz, 1974) that measures both cluster distance and compactness. The normalized estimate of the amount of color in each cluster is measured by the weight for the Gaussians in the expectation maximization step in the blind color decomposition. The optimal parameters are selected experimentally by optimizing the quantitative measure of cluster distance, compactness, and amount of color in each cluster. Finally, the visual quality of the stain, and in particular the nuclear texture was evaluated by two independent expert uropathologists on a scale from 1 to 5, with 5 indicating the best texture.

### Segmentation Algorithm

The objective of the segmentation is to find the glandular boundaries, in order to identify glandular architectural changes linked to malignancy. The segmentation of prostate glands is made difficult by tightly packed glands where boundaries often touch, and by highly irregularly shaped glands where it is often hard to discern where the glands start and end (illustrated by the fused gland in Figure 1F).

**Figure 4:**
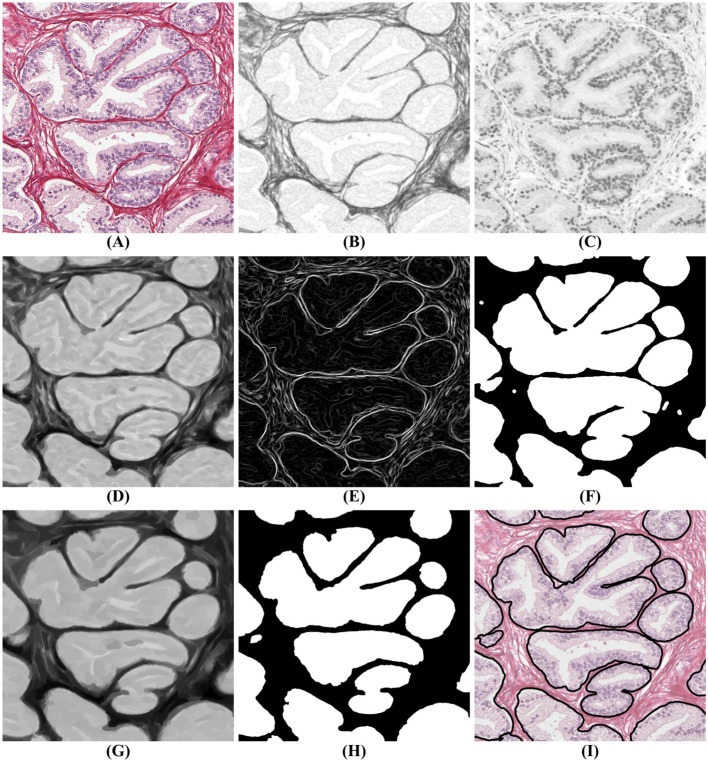
Gland segmentation steps. **(A)** original image; **(B)** stromal tissue; **(C)** epithelial tissue; **(D)** morphological opening step; **(E)** gradient of the stromal tissue; **(F)** glandular mask; **(G,H)** seeds extraction; **(I)** final result obtained by applying a watershed with seeds **(H)** and mask **(F)** on the original image **(A)**.

**Figure 5:**
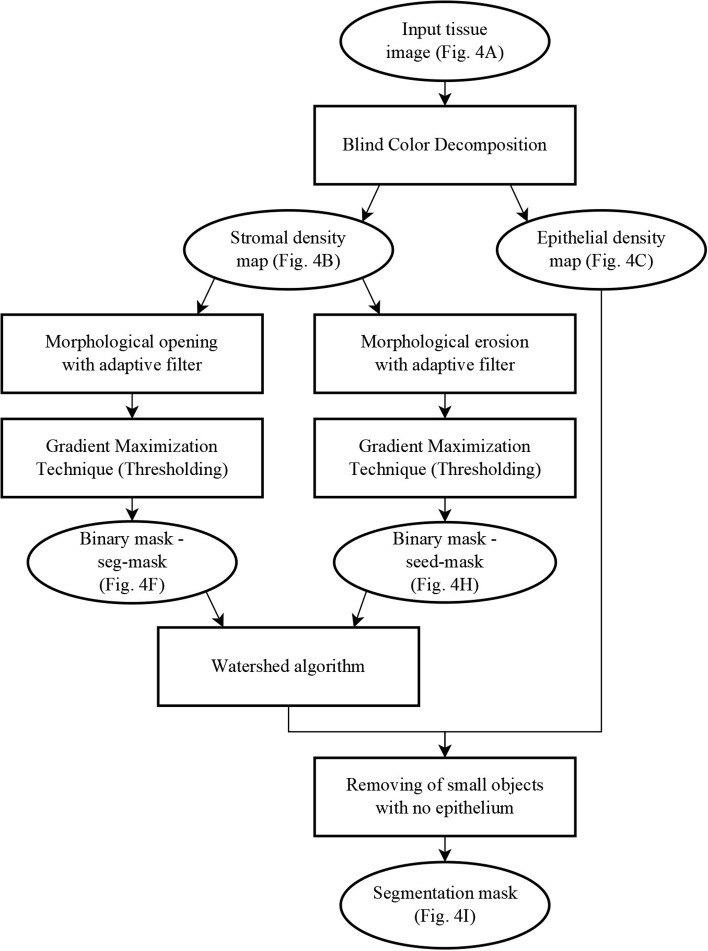
Flow chart of the segmentation algorithm, where elliptical boxes represent data and rectangular boxes represent each one of the algorithm steps.

The segmentation algorithm proceeds in two steps: first, a mask identifies clusters of glands with adjoining boundaries. Second, we find one seed per gland in these clusters and grow these seeds until they meet the mask or other glands using a watershed technique (Beucher and Lantuéj, 1979). The BCD algorithm described earlier decomposes the tissue image (illustrated in Figure 4A) into a stromal density map (Figure 4B) and an epithelial density map (Figure 4C). The stromal density map is the basis for the segmentation, while the epithelial density map is used only to remove small objects that do not constitute complete glands. Figure 5 shows the algorithm's flow chart, where elliptical boxes represent data and rectangular boxes represent each one of the algorithm steps.

Starting with the stromal density map, morphological opening smooths the boundaries of the glands (Figure 4D). From the resulting image, gradient maximization thresholding (Landini et al., 2017) gives us a binary segmentation-mask covering the gland clusters. This thresholding technique has the advantage that it uses the local image content to find an optimal threshold rather than basing the threshold only on the global image histogram (Figures 4E–F).

The next step is to find one seed for each gland. The seeds are obtained by eroding the stromal density map (Figure 4G) and by creating a binary image, again using gradient maximization thresholding (Figure 4H). The erosion will separate glands that are weakly connected, that is connected by only a few pixels. Since some glands may touch over multiple pixels, a circular structuring element for the erosion may either be too small to separate glands or so big that it would remove small glands. Thus, we use tensor-based elliptical structuring elements (Landström and Thurley, 2013) that adapt to lines in regions of strong single-directional features and to disks where the tissue has no prevalent direction. An adaptive filter, which varies depending on the local image structure, ensures correct separation of distinct glands without removing the small glands.

In the final step of the segmentation we apply a watershed algorithm initialized with the seeds (Figure 4H) and with the segmentation-mask (Figure 4F) as boundary. Note that a couple of individual glands are connected in the resulting image. This is because they are connected by more pixels than the kernel size of the erosion. Do notice that the choice of kernel is a compromise between a large a kernel that would remove small glands, and a small a kernel that would not separate enough glands.

In the resulting segmentation, there are always some small objects which may be glands or may be other objects, such as pieces of stroma, that should be removed. As all glands must contain at least one nucleus, we remove objects without nuclei. This is accomplished by referring to the corresponding region in the epithelial density map and determining whether there are sufficient pixels of high intensity value indicating the presence of a nucleus. The final result of the glandular segmentation is shown in Figure 4I.

This algorithm relies on a few parameter settings:

- The size of the kernel for the morphological opening depends on the amount of stroma between pairs of glands needed for their separation. A large kernel will tend to segment more distinct glands while a smaller kernel will tend to group glands together. A kernel size of 6 × 6 pixels appears to be a good compromise for our data.
- The minimum size of an object is set to the mean size of a epithelial nucleus in our dataset, that is 90 pixels.
- The minimum intensity of the nuclei in the epithelial density map is fixed to 0.7, where pixel intensities vary from 0 (black) to 1 (white).

## Results

An expert pathologist checked the quality of the segmentation on 486 images, 1,500 by 1,000 pixels, chosen from prostatectomies. The expert classified 11,447 objects from these images into four categories: glandular objects (correct segmentation) (vessels, nerves and groups of lymphocytes); under-segmentation (only part of a gland correctly segmented); and over-segmentation (more than the gland is identified as one). The results are presented in Table 1, with the objects categories and the number of objects in each category. The gland categories include discrete well-formed glands, poorly formed, cribriform, and fused glands, glomeruloid structures, intraductal carcinoma, individual cells and files.

**Table 1:** Categorization of 11,447 objects automatically segmented from 486 images into glands with their Gleason Grade (GG) and other objects.

**Category**	**Number of objects**	**%**
Benign glands	1,198	10,47
PIN glands	121	1,06
GG 3: Well-formed glands	3,505	30,62
GG 4: Poorly-formed glands	2,278	19,90
GG 4: Fused glands	112	0,98
GG 4: Cribriform glands	44	0,38
GG 4: Glomeruloid structures	11	0,10
Intraductal carcinoma	24	0,21
GG 5: Individual cells and files	476	4,16
Over-segmentation (object segmented with stroma)	397	3,47
Under-segmentation (object partially segmented)	494	4,32
Non-glandular objects (correctly segmented nerves, vessels, stromal cells, *etc*.)	2,787	24,35
Total	11,447	100,00

These results show that 92% of the objects found are accurately segmented, of which 74% are glands and 26% are correctly segmented non-glandular objects (vessels, nerves, lymphocytes, or stromal tissue). These non-glandular objects are removed during classification, as their intrinsic structures differ from normal glands (different shape of nuclei, different shape of the object, no lumen, etc.).

**Figure 6:**
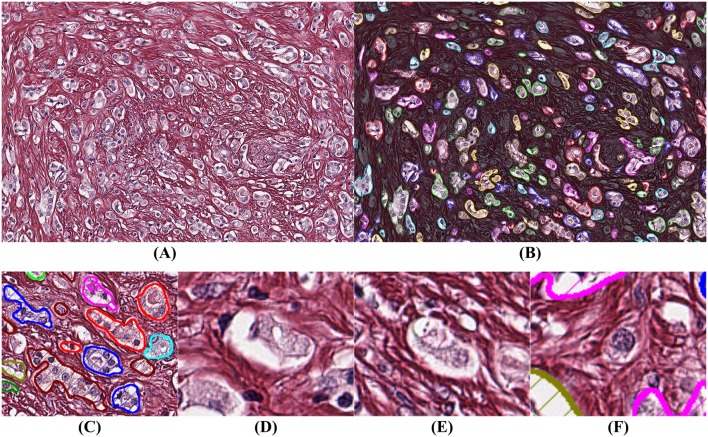
Example of segmentation on high Gleason grade tissue (grade 4 and 5). The background has been darkened in **(B)** to make the glandular segmentation visible; **(C)** close-up on gland segmentation from **(A)**; **(D–F)** examples of missed glands: **(D,E)** glandular structures without visible nuclei; **(F)** nuclei without surrounding epithelium.

It is more difficult to obtain ground truth for the number of missed glands in the segmentation process. Missed glands occur mostly in areas with aggressive cancer, when glands are small and not well-formed. In order to quantify the number of missed glands, a high-grade case of prostatic cancer was selected, segmented, and analyzed (Figure 6).

By comparing Figures 6A,B we observe that the segmentation is highly accurate. By assuming that a gland must have at least one epithelial nucleus and some epithelial cytoplasm, as seen in Figure 6C, our algorithm rejects structures without a nucleus as shown in Figures 6D,E, and structures without epithelial cytoplasm as shown in Figure 6F.

In summary, gland segmentation that relies on PSR-Htx accurately identifies the boundary between the stroma and the epithelium in most instances. To our knowledge this is the first algorithm that automatically identifies glands of all grades, including benign glands, PIN, well-formed glands (GG 3), poorly formed glands (GG 4), fused glands (GG 4), cribriform glands (GG 4), glomeruloid structures, intraductal carcinoma, individual cells, and files (GG 5).

## Discussion

### Applications of the Segmentation Algorithm

**Figure 7:**
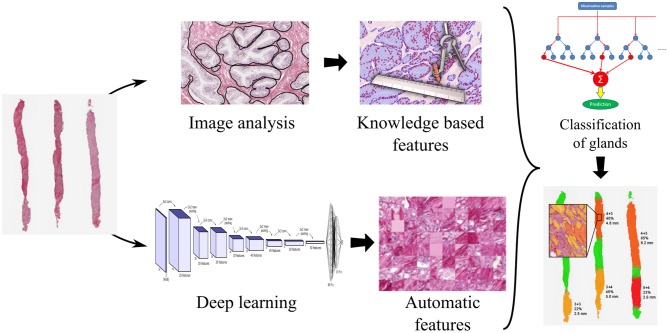
Processing pipeline for a decision support system for prostate cancer malignancy grading, with prostate biopsy sections as input and an overlay map displaying grades on glands as output.

Glandular segmentation of prostate tissue is key to building a detailed ground truth data set for machine vision and deep learning automatic grading algorithms. An accurate segmentation algorithm facilitates easy ground truth labeling on a glandular level by facilitating the presentation of the glands one-by-one to the pathologist. This method was used to label 11,447 glands. From the segmented glands it is possible to extract glandular features known to be linked to malignancy (size of glands, roundness of glands, etc.) to train a classifier. We have found that the optimal classification results from combining machine and deep learning, trained on the glandular architecture and the glandular features. Please refer to Figure 7.

Glandular segmentation is also the foundation for a malignancy grading decision support system enabling accurate grading and also the measure of the amount of cancer in a tissue sample. The segmentation makes it possible to create color-coded overlays corresponding to the grade of individual glands, green for benign, yellow for grade 3, orange for grade 4, and red for grade 5, as is illustrated in Figure 7. The color overlays make it easy for the pathologist to locate the cancer regions and to verify their correctness.

### Computational Complexity

Histopathological images are large, up to 100,000 by 100,000 pixels, making it imperative that each step in the grading pipeline be as efficient as possible. All algorithms in this paper are fast and highly parallelizable, as they rely on well-known image analysis methods for which there are optimized implementations in the literature. Adaptive mathematical morphology, watershed segmentation, and thresholding can all be executed on many-core or multi-core architecture, such as GPUs or computation grids. The blind color decomposition used to separate images into density maps is the most time-consuming part of the pipeline. But this step can be optimized by learning the absorption and noise models once for the tissue stained with PSR-Htx and applying this model in parallel to decompose the image in the grading pipeline.

### Does the Glandular Structure Determine the Grade?

The most interesting and surprising result is seen in Figures 3C,D, which shows that PSR-Htx can identify glandular structures that do not appear to be present in the same tissue stained with H&E. That means that the PSR-Htx-stained tissue would be given a lower grade, while the H&E-stained tissue would be given a higher grade. That begs the question: is the grade determined by the true glandular structure or by what is visible with H&E, as it was in the time of Gleason?

## Ethics Statement

This study was carried out in accordance with the recommendations of the Central Ethic Review Board of Sweden. All subjects gave written informed consent in accordance with the Declaration of Helsinki. The protocol was approved by the Uppsala Section of the Central Ethical Board of Sweden (EPN nr 2006/101/3, #214;25-2006).

## Disclosure

The work herein is partially covered under two issued patents and two pending patents. Two authors (IC) and (CA), have a financial interest in CADESS Medical AB. Some parts of this paper were presented as a poster at ECDP 2018.

## Author Contributions

CA designed and implemented most of the algorithms. He also wrote parts of the manuscript. AT is the expert that examined the over 20,000 prostate glands for accuracy. She also provided expertise about prostate cancer diagnosis including malignancy grading. AD selected the histopathological sections for the study and helped design the stain protocol. IC is the PI, and planned all aspects of the project, contributed to the stain evaluation, the color decomposition, and wrote most of the manuscript.

### Conflict of Interest Statement

The authors declare that the research was conducted in the absence of any commercial or financial relationships that could be construed as a potential conflict of interest.
